# PD-L1, FGFR1, PIK3CA, PTEN, and p16 expression in pulmonary emphysema and chronic obstructive pulmonary disease with resected lung squamous cell carcinoma

**DOI:** 10.1186/s12890-019-0913-8

**Published:** 2019-09-03

**Authors:** Ken Arimura, Yasuo Sekine, Kenzo Hiroshima, Satoru Shimizu, Noriyuki Shibata, Mitsuko Kondo, Kiyoshi Takeyama, Etsuko Tagaya

**Affiliations:** 1 Department of Respiratory Medicine, Tokyo Women’s Medical University, Tokyo, Japan; 2 Departments of Thoracic Surgery, Tokyo Women’s Medical University Yachiyo Medical Center, Chiba, Japan; 3 Departments of Pathology, Tokyo Women’s Medical University Yachiyo Medical Center, Chiba, Japan; 4 Departments of Medical Education, Tokyo Women’s Medical University School of Medicine, Tokyo, Japan; 5 Human and Experimental Pathology and Neuroscience, Tokyo Women’s Medical University School of Medicine, Tokyo, Japan

**Keywords:** PD-L1, Lung squamous cell carcinoma, Emphysema, COPD

## Abstract

**Background:**

Emphysema and chronic obstructive pulmonary disease (COPD) are well known independent risk factors for lung cancer. However, the developmental mechanisms between emphysema/COPD and lung cancer remain unknown. The purpose of this study was to evaluate PD-L1, FGFR1, PIK3CA, PTEN, and p16 expression in squamous cell carcinoma (SCC) associated with emphysema/COPD.

**Methods:**

A total of 59 patients with squamous cell lung carcinoma (SCC) resected between 2008 and 2012 were retrospectively reviewed. Emphysema was assessed according to the Goddard score. Total severity was divided into none-mild (0–7), moderate (8–15), and severe (≥ 16). Local severity around the existing tumor was divided into no emphysema (0) and presence of emphysema (1–4). COPD severity was based on the Global Initiative for Chronic Obstructive Lung Disease (GOLD) criteria. PD-L1, FGFR1, PIK3CA, PTEN, and p16 expression were evaluated by immunohistochemistry (IHC). Expression level was classified as tumor cells (TC) 3 (≥ 50%), TC2 (5–49%), TC1 (1–4%), or TC0 (< 1%), and as tumor-infiltrating immune cells (IC) 3 (≥ 50%), IC2 (5–49%), IC1 (1–4%), or IC0 (< 1%) for PD-L1. Expression level was compared between none-mild/moderate-severe total emphysema, no/presence of local emphysema, no COPD/COPD, and GOLD 1/GOLD 2, 3.

**Results:**

PD-L1 expression was significantly correlated with severity of emphysema in TC0, 1, 2 vs. TC3 (*P* = 0.012). PD-L1 was significantly higher inversely in none-mild emphysema compared to moderate-severe (95% CI, 0.061–5.852, *P* = 0.045). There were no other significant associations between PD-L1, FGFR1, PIK3CA, PTEN, and p16 expression and total/local severity of emphysema or presence of COPD/GOLD stage.

**Conclusions:**

PD-L1 expression in SCC was correlated with severity of emphysema in TC0, 1, 2 vs. TC3 and more frequent in none-mild emphysema than moderate-severe emphysema.

## Background

Lung cancer is the primary cause of cancer-related death worldwide. It is well known that smoking is a major risk factor for lung cancer [1]. Smoking causes emphysema and chronic obstructive pulmonary disease (COPD) [2, 3]. Several studies have found that emphysema and COPD are independent risk factors for lung cancer [4–6], especially squamous cell lung carcinoma (SCC) [7, 8].

The efficacy of immune checkpoint inhibitors has been established for SCC that express programmed death ligand 1 (PD-L1) [9, 10]. Although targeted therapy against adenocarcinoma with epidermal growth factor receptor (EGFR) gene mutation or anaplastic lymphoma kinase (ALK) and ROS1 proto-oncogene receptor tyrosine kinase (ROS1) rearrangements have shown dramatic effects [11–13], few targeted therapies against SCC have been identified. Previous reports have suggested that potential targets for therapy against SCC including fibroblast growth factor receptor 1 (FGFR1), phosphatidylinositol - 4,5 - bisphosphate 3 - kinase catalytic subunit alpha (PIK3CA), phosphatase and tensin homolog (PTEN), and p16 [14–17].

The genetic causes of emphysema and COPD are largely unknown [18, 19]. Furthermore, the developmental pathway between COPD and non-small cell lung cancer (NSCLC) remains elusive [20–22]. The purpose of this study was to evaluate PD-L1, FGFR1, PIK3CA, PTEN, and p16 expression in SCC associated with emphysema and COPD.

## Methods

### Ethical considerations

This was a retrospective study approved by the institutional review board (Date of approval: Dec 27, 2012, approval number: 2693) of Tokyo Women’s Medical University Hospital. Written informed consent was obtained from all patients before tumors were resected and additional consent was waived.

### Patients population and samples

This study included 59 patients who underwent surgery for SCC between February 2008 and December 2012 at Tokyo Women’s Medical University, Yachiyo Medical Center. All patients had computerized tomography (CT) and respiratory function tests to assess the suspected malignancy and determine eligibility for surgical intervention prior to surgery. Resected tumors were immediately fixed with 10% formalin, stained with hematoxylin and eosin (HE) and prepared for immunohistochemistry (IHC) staining.

### Definition of emphysema

The severity of emphysema on CT was visually assessed by two independent pulmonologists according to the Goddard scoring system [23, 24]. Low attenuation area (LAA) on six images of three lung slices (the right and left lungs were evaluated separately) were analyzed for each patient [25]. Each image was scored as follows: score 0 (no LAA), score 1 (LAA of 1–25%), score 2 (LAA of 26–50%), score 3 (LAA of 51–75%), and score 4 (LAA of ≥76%) [23]. Total severity score was categorized into one of three groups as follows: none-mild (0–7), moderate (8–15), and severe (≥ 16). Local severity score around the tumor was classified as no-emphysema (0) and presence of emphysema (1–4).

### Definition of COPD

Respiratory function tests were performed to assess the eligibility for surgery. Diagnosis of COPD was based on the Global Initiative for Chronic Obstructive Lung Disease (GOLD) criteria after confirming the presence of persistent airflow limitation by a post-bronchodilator forced expiratory volume in one second (FEV1)/forced vital capacity (FVC) < 70% [26]. The severity of air flow limitation was classified using the GOLD staging criteria with % predicted FEV1, as follows: GOLD 1 (≥ 80%), GOLD 2 (50–79%), GOLD 3 (30–49%), and GOLD 4 (< 30%) [26].

### Molecular and IHC analysis

PD-L1, FGFR1, PIK3CA, PTEN, and p16 expression was evaluated by IHC staining of archived resected samples. IHC was performed with the following antibodies: PD-L1 (clone SP-142, diluted 1:100; Ventana Medical System, Arizona, USA), FGFR-1 (polyclonal, diluted 1:400; Abcam, Cambridge, UK), PIK3CA (Recombinant, diluted 1:400; Abcam), PTEN (clone 138G6, diluted 1:200; Cell Signaling, Massachusetts, USA), and p16 (Recombinant, diluted 1:2000; Abcam).

To improve the expression, the tissues were pre-treated with microwaves in ethylenediaminetetraacetic acid for PD-L1, or in retrieval solution (DAKO Target Retrieval Solution pH 9, Glostrup, Denmark) for FGFR1 in citrate buffer for PTEN and p16, before staining. Positive controls in IHC protocols were specimens from pulmonary SCC where the target molecules have been confirmed to be positive. To validate the specificity of the secondary antibody, negative reaction control sections obtained by omission of the primary antibody were used. IHC expression was evaluated by one experienced pathologist and pulmonologist in a blinded fashion. Each expression of tumor cells was classified by staining as follows: tumor cells (TC) 3 (≥ 50%), TC2 (5–49%), TC1 (1–4%), and TC0 (< 1%) [27]. Furthermore, tumor-infiltrating immune cells were also evaluated for PD-L1 as follows: immune cells (IC) 3 (≥ 50%), IC2 (5–49%), IC1 (1–4%), and IC0 (< 1%). Immune cells were evaluated only for PD-L1, based on a previous study [27]. If the score did not initially agree between evaluators, a discussion was initiated. Analysis of PD-L1 expression was as follows: TC0 vs. TC1, 2, 3, TC0, 1 vs. TC 2, 3, TC0, 1, 2 vs. TC3 and IC0 vs. IC1, 2, 3, IC0, 1 vs. IC 2, 3, IC0, 1, 2 vs. IC3. Analysis of FGFR1, PIK3CA, PTEN, and p16 expression was as follows: TC0 vs TC1, 2, 3.

## Data analysis

Each expression of IHC was compared between none-mild (0–7) and mild-severe (8–24) emphysema groups of total Goddard scoring, no-emphysema (0) and presence of emphysema (1–4) tumor area groups, non-COPD and COPD groups, and GOLD 1 and GOLD 2, 3 groups. Data analysis was carried out using Statistical Analysis System (SAS institute, Cary, NC) and Graph Pad PRISM (GraphPad Software, La Jolla, CA). *P* values < 0.05 were regarded as statistically significant. The *t*-test was used to compare between each expression and Goddard score. Chi-squared test was used to compare between each expression and severity of emphysema, presence of COPD/Gold staging. Groups were compared using 95% confidence intervals (CI).

## Results

### Baseline characteristics and molecular classification by staining

**Fig. 1:**
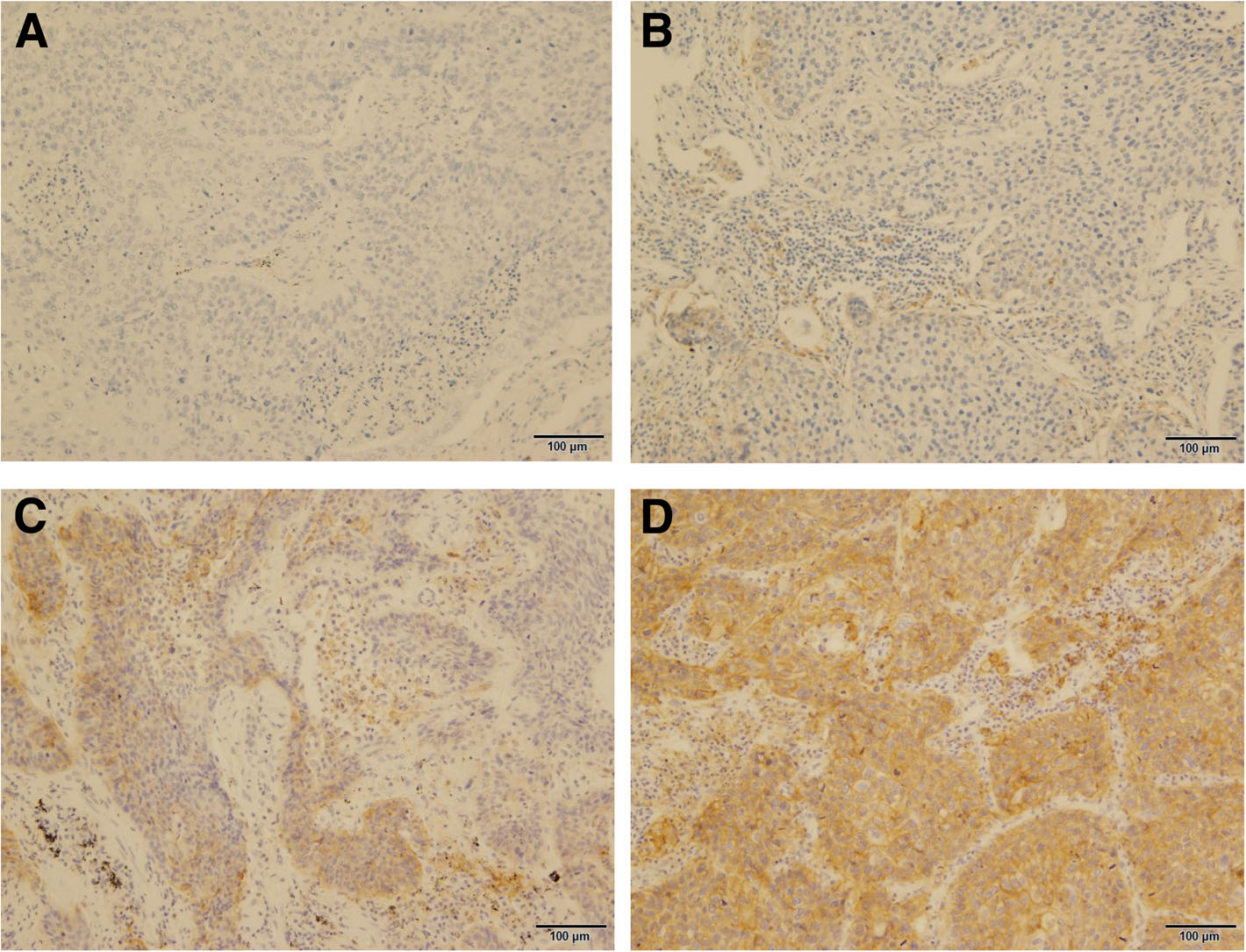
Representative image of PD-L1 expression for each staining level (10X). A TC0 and IC0 (< 1%), B TC1 and IC1 (1–4%), C TC2 and IC2 (5–49%), D TC3 and IC3 (50–100%). *PD-L1* programmed death ligand 1, *TC* tumor cells, *IC* immune cell

A total of 59 patients with surgically resected SCC were eligible for this study. Patient baseline characteristics included sex, median age, smoking history, histology, pathological stage, total severity of emphysema, local severity of emphysema, presence of COPD, and GOLD stage (Table 1). There were no significant differences between total emphysema status, local emphysema status, COPD status and GOLD stage based on sex, smoking history and stage (Table 2). Classifications of TC0–3, IC0–3 for PD-L1 (Fig. 1) and of TC0–3 for FGFR1, PIK3CA, PTEN, and p16 expression were recorded (Table 3).

**Table 1:** Patient characteristics

Patient Characteristics	No (%)
Patients	59
Median age (range)	72.7 (56–85)
Sex	
Male	50 (84.7)
Female	9 (15.3)
Smoking history	
Ever	57 (96.6)
Never	2 (3.4)
Median pack year (range)	58.9 (0–184)
Histology	
Squamous cell carcinoma	59 (100)
Pathological stage	
I	38 (64.4)
II	16 (27.1)
III	4 (6.8)
recurrence	1 (1.7)
Severity of emphysema	
mild	40 (67.8)
moderate	14 (23.7)
severe	5 (8.5)
Local severity of emphysema	
non	23 (39.0)
presence	36 (61.0)
Presence of COPD	
No COPD	29 (49.2)
COPD	30 (50.8)
Staging of GOLD	
GOLD1	37 (62.7)
GOLD2	21 (35.6)
GOLD3	1 (1.7)

*COPD* chronic obstructive pulmonary disease, *GOLD* Global Initiative for Chronic Obstructive Lung Disease

**Table 2:** Comparison of patient characteristics based on total emphysema, local emphysema, COPD, and GOLD stage

Factors	none-mild	moderate-severe	*P* value	No Local emphysema	Local emphysema	*P* value	No COPD	COPD	*P* value	GOLD1	GOLD2,3	*P* value
SEX												
Male	33	17	0.78	18	32	0.54	26	24	0.59	34	16	0.14
Female	7	2		5	4		3	6		3	6	
Smoking												
Never	2	0	0.61	2	0	0.2	2	0	0.34	2	0	0.54
Ever	38	19		21	36		27	30		35	22	
Stage												
I	27	11	0.63	16	22	0.84	21	17	0.62	28	10	0.17
II	10	6		5	11		7	9		7	9	
III	3	1		2	2		1	3		2	2	
recurrence	0	1		0	1		0	1		0	1

**Table 3:** Classification of each expression

Classify of expression (%)	PD-L1	FGFR1	PIK3CA	PTEN	p16
TC0	7 (11.9)	26 (44.1)	55 (93.2)	35 (59.3)	22 (37.3)
TC1	12 (20.3)	15 (25.4)	3 (5.1)	8 (13.6)	19 (32.2)
TC2	17 (28.8)	17 (28.8)	1 (1.7)	15 (25.4)	16 (27.1)
TC3	23 (39.0)	1 (1.7)	0 (0)	1 (1.7)	2 (3.4)
IC0	1 (1.7)				
IC1	2 (3.4)				
IC2	25 (42.4)				
IC3	31 (52.5)				

*PD-L1* programmed death ligand 1, *FGFR1* fibroblast growth factor receptor 1, *PIK3CA* phosphatidylinositol-4,5-bisphosphate 3-kinase catalytic subunit alpha, *PTEN* phosphatase and tensin homolog, *TC* tumor cells, *IC* immune cell

### Comparison between each classification of expression and severity of emphysema

**Fig. 2:**
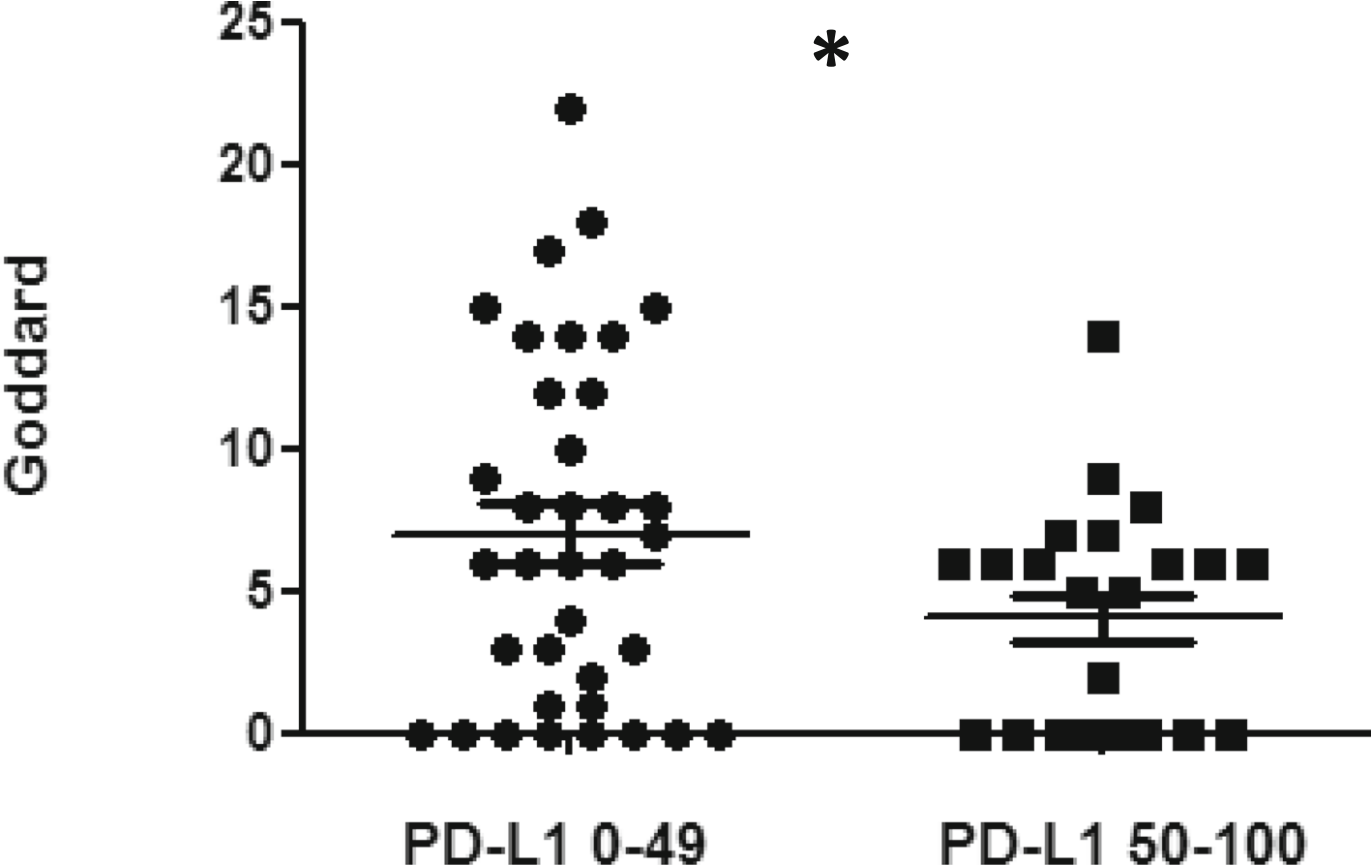
Comparison between TC0, 1, 2 (PD-L1 0–49) and TC3 (PD-L1 50–100) based on Goddard score PD-L1 expression was more than 50% when Goddard score was low (95% CI: 0.061–5.852, *P = 0.045). *PD-L1* programmed death ligand 1

IHC expression of each molecular antibody was compared to total/local severity of emphysema (Table 4). PD-L1 expression was significantly correlated with total severity of emphysema in TC0, 1, 2 vs. TC3 (*P* = 0.012). However, there was no other significant association between classification of expression and total/local severity of emphysema. Since there was significant association in total severity of emphysema, the results between TC0, 1, 2 (PD-L1 0–49) and TC3 (PD-L1 50–100) for total severity of emphysema were compared. There was significant difference between groups (95% CI: 0.061–5.852, *P* = 0.045) (Fig. 2).

**Table 4:** Comparison between each classification of expression and severity of emphysema, presence of COPD, GOLD staging

	Total severity of emphysema			Local severity of emphysema			Presence of COPD			Staging of GOLD		
Classify of expression	none-mild	moderate-severe	*P* value	none	present	*P* value	No COPD	COPD	*P* value	GOLD1	GOLD2,3	*P* value
PD-L1												
TC0	6	1	0.28	5	2	0.06	3	4	0.72	5	2	0.61
TC1,2,3	34	18		18	34		26	26		32	20	
TC0,1	14	5	0.5	9	10	0.36	7	12	0.19	11	8	0.6
TC2,3	26	14		14	26		22	18		26	14	
TC0,1,2	20	16	0.012	13	23	0.57	17	19	0.71	22	14	0.75
TC3	20	3		10	13		12	11		15	8	
IC0	1	0	0.49	1	0	0.2	0	1	0.32	1	0	0.44
IC1,2,3	39	19		22	36		29	29		36	22	
IC0,1	2	0	0.32	2	0	0.07	0	2	0.16	2	0	0.27
IC2,3	38	19		21	36		29	28		35	22	
IC0,1,2	20	7	0.34	14	13	0.06	16	11	0.15	18	9	0.56
IC3	20	12		9	23		13	19		19	13	
FGFR1												
TC0	17	9	0.72	8	18	0.25	13	13	0.91	14	12	0.21
TC1,2,3	23	10		15	18		16	17		23	10	
PIK3CA												
TC0	36	19	0.15	22	33	0.55	28	27	0.32	33	22	0.11
TC1,2,3	4	0		1	3		1	3		4	0	
PTEN												
TC0	23	12	0.68	17	18	0.07	16	19	0.52	20	15	0.29
TC1,2,3	17	7		6	18		13	11		17	7	
p16												
TC0	13	9	0.27	10	12	0.43	14	8	0.09	12	10	0.32
TC1,2,3	27	10		13	24		15	22		25	12	

*COPD* Chronic obstructive pulmonary disease, *GOLD* Global Initiative for Chronic Obstructive Lung Disease, *PD-L1* Programmed death ligand 1, *TC* Tumor cells, *IC* Immune cells, *FGFR1* Fibroblast growth factor receptor 1, *PIK3CA* Phosphatidylinositol-4,5-bisphosphate 3-kinase catalytic subunit alpha, *PTEN* Phosphatase and tensin homolog

### Comparison between each classification of expression and presence of COPD/ GOLD stage

The results of each classification of expression and presence of COPD/Gold stage was compared (Table 4). There was no significant association between classification of expression and presence of COPD/GOLD stage.

## Discussion

Few reports have investigated the associations between TC, PD-L1, emphysema, mutational analysis, and COPD in NSCLC [28, 29]. One study on lung adenocarcinoma found that patients with emphysema had more PD-L1 positive cells than patients without emphysema [28]. Another study on NSCLC showed that PIK3CA expression was significantly associated with COPD [29].

In this study, PD-L1 expression was significantly correlated with severity of total emphysema in TC0, 1, 2 vs. TC3 (*P* = 0.012). Furthermore, there was a significant difference inversely in total severity of emphysema between TC0, 1, 2 (PD-L1 0–49) and TC3 (PD-L1 50–100) (95% CI: 0.061–5.852, *P* = 0.045). There were no other significant associations between classification of expression and total/local severity of emphysema, and between classification of expression and presence of COPD/GOLD stage. These results differ from previous studies on the associations between PD-L1 expression and emphysema in patients with lung adenocarcinoma [28], and between PIK3CA gene mutation and COPD in patients with NSCLC [29]. One explanation for this discrepancy is that our study focused on SCC while other studies focused on lung adenocarcinoma or NSCLC. Another is that our study focused on protein expression while other study focused on the gene mutation. These differences may have influenced the results and may account for the differences in PD-L1 expression and PIK3CA.

This study identified for the first time a significant association between high expression of PD-L1 (≥ 50%) and none-mild emphysema in SCC. However, the mechanism behind this association remains unknown. Previous studies have shown an increased proportion of PD-1 positive CD8 T-cells in the lungs of patients with COPD lung such as lung emphysema. This appeared to contribute to dysfunctional of CD8 T-cells [30, 31]. Our findings suggest that tumor need not escape from CD8 T-cell attack, particularly given the low expression of PD-L1 when emphysema is moderate-severe. On the contrary, if a tumor escapes CD8 T-cell attack, it might be attributed to high expression of PD-L1 in none-mild emphysema.

This study found that none-mild emphysema was an independent predictive factor of high PD-L1 expression (≥ 50%). This result suggests the utility of non-invasive ways of predicting high expression of PD-L1 (≥ 50%), such as CT imaging. CT imaging may also be an effective way of deciding to use immune checkpoint inhibitors [9, 10] in patients with SCC before determining PD-L1 expression with IHC. This might prevent significant life threatening events associated with surgery and bronchoscopy, such as decreasing respiratory function, embolism, bleeding, and pneumothorax. In addition, this might lead to insights regarding the mechanistic correlation between PD-L1 expression and emphysema.

This study had several limitations. First, this was a retrospective study performed at a single institution, which may have influenced the results. A prospective multicenter randomized control trial is needed to confirm our results. Second, although this study shows an association between each expression and Goddard, COPD, and GOLD stage, the associations between those and overall survival rates remain unknown. An evaluation of overall survival is needed. Third, although LAA was evaluated with Goddard criteria, it is unclear if the automated evaluation for LAA [32] would provide the same results. Additional studies are needed to determine what methods are most appropriate for evaluating LAA.

## Conclusion

In conclusion, there was a significant association between TC0, 1, 2 vs. TC3 groups and none-mild vs. moderate-severe emphysema groups in patients who underwent surgical resection of lung SCC. PD-L1 expression was significant higher in none-mild emphysema than in moderate-severe emphysema. There was no significant association between any other classification of expression for PD-L1, FGFR1, PIK3CA, PTEN, p16 and total/local severity of emphysema, between classification of expression for PD-L1, FGFR1, PIK3CA, PTEN, p16 and presence of COPD/GOLD staging. Further studies are needed to evaluate overall survival between classification of each expression and total/local severity of emphysema, presence of COPD/GOLD stage.

## Data Availability

The dataset supporting the conclusions of this study is presented in this manuscript. The clinical detail dataset is available with author and corresponding author, not publicly available.

## Abbreviations

COPD: Chronic obstructive pulmonary disease
GOLD: Global Initiative for Chronic Obstructive Lung Disease
PD-L1: Programmed death ligand 1
SCC: Squamous cell carcinoma
